# Photosynthetic Characterization of Oil Palm (*Elaeis guineensis* Jacq.) Seedlings During Late In Vitro Development and Acclimatization

**DOI:** 10.3390/plants14091299

**Published:** 2025-04-25

**Authors:** Rodrigo Andrés Avila-Diazgranados, Wilmer Tezara, Hernán Mauricio Romero

**Affiliations:** 1 Oil Palm Biology and Breeding Research Program, Colombian Oil Palm Research Center-Cenipalma, Calle 98 70-91, Bogota 111121, Colombia; ravila@cenipalma.org; 2 Estación Experimental Mutile, Facultad de Ciencias Agropecuarias, Universidad Técnica Luis Vargas Torres, Esmeraldas 080102, Ecuador; wilmer.tezara@ciens.ucv.ve; 3 Instituto de Biología Experimental, Universidad Central de Venezuela, Apartado 47114, Caracas 1041-A, Venezuela; 4 Department of Biology, Universidad Nacional de Colombia, Carrera 30 Calle 45, Bogota 111311, Colombia

**Keywords:** in vitro ecophysiology, micropropagation, gas exchange, acclimation, plant tissue culture

## Abstract

Oil palm (*Elaeis guineensis* Jacq.) is the leading global oil-producing crop due to its high oil yield. Increasing global demands for palm oil require efficient propagation. Conventional breeding is practical but slow, making micropropagation an attractive alternative for rapidly multiplying superior genotypes. However, transitioning from in vitro to ex vitro conditions causes physiological stress, restricting survival and productivity. This study assessed gas exchange and chlorophyll fluorescence dynamics during acclimatization from in vitro conditions to field establishment, comparing the seedlings obtained in vitro with conventional seed-derived palm seedlings to conventional seed-derived palms. A pronounced photosynthetic efficiency decline occurred after transfer from in vitro culture, followed by a gradual recovery. The photosynthetic rate (*A*) increased from 0.86 µmol m^−2^ s^−1^ early in acclimatization to 15.43 µmol m^−2^ s^−1^ in field-established seedlings. Physiological characterization using CO_2_ and light response curves identified the reductions in carboxylation efficiency and overall quantum yield CO_2_. These biochemical constraints gradually diminished during acclimatization, facilitating a transition from heterotrophic to autotrophic growth. Chlorophyll fluorescence analysis revealed remarkable photoinhibition during initial ex vitro stages, indicated by a decreased maximum quantum efficiency of photosystem II. However, the seedlings progressively restored photochemical function throughout subsequent acclimatization phases. These findings highlight the importance of carefully regulating environmental parameters—particularly irradiance, humidity, and carbon availability—during early seedling acclimatization. The effective management of growth conditions significantly mitigates physiological stress, ensuring robust photosynthetic activity and optimized stomatal regulation. The improved acclimatization practices, therefore, can substantially enhance seedling survival rates, physiological resilience, and the overall field performance of micropropagated oil palms. Future research should focus on refining acclimatization protocols, emphasizing targeted physiological interventions to maximize the efficiency, commercial viability, and sustainability of oil palm clonal propagation.

## 1. Introduction

The clonal propagation of oil palm (*Elaeis guineensis* Jacq.) allows the selection of elite or genetically outstanding genotypes with superior yields and high oil quality [1,2,3]. Since oil palms have high heterozygosity, conventional breeding requires extensive evaluation periods that may exceed 15 years, making micropropagation an efficient alternative for developing commercial plantations with elite clones [2].

Environmental conditions such as low temperature variations, high relative humidity, and controlled photoperiods are necessary for seedling development during in vitro propagation [4]. However, these conditions often lead to morphoanatomical alterations in seedlings compared to those generated from seeds, making them more sensitive to moisture loss during the hardening process [5]. Understanding how these environmental effects impact clonal material is essential as they influence growth and metabolism and produce bioactive compounds [6].

The internal environment of in vitro culture flasks, characterized by high relative humidity, variable CO_2_ concentrations, and the potential accumulation of ethylene and other gases, influences plants’ anatomical, physiological, and morphological characteristics. These factors can cause plant losses during acclimatization [7,8]. Micropropagated seedlings frequently exhibit low survival rates due to these anatomical and physiological disorders [8]. Therefore, successful acclimatization is required for commercial micropropagation [9]. Although seedlings exhibit low photosynthetic capacity at the beginning of the acclimatization process, high humidity conditions must be maintained to prevent excessive water loss through the stomata and to aid in photosynthetic recovery [10].

The low photosynthetic capacity in early acclimatization stages is primarily attributed to the effects of in vitro conditions on leaf anatomy and physiology. Photosynthesis reduction has been linked to decreased activity or rubisco content [11]. Asmar et al. [12] highlighted that despite the advantage of micropropagation in generating a large number of seedlings, the heterotrophic conditions of in vitro culture can lead to reduced epicuticular wax deposition, poor mesophyll differentiation, rudimentary vascular bundles, and limited stomatal control. Changes in leaf tissue morphology occur during acclimatization, particularly in stomatal density and function, due to increased light intensity and decreased relative humidity. The most significant alterations include a decrease in net photosynthetic rate (*A*) during the initial days ex vitro, accompanied by an increased transpiration rate (*E*) and stomatal conductance (*g_s_*) [9,13,14,15].

Since the 1970s, the micropropagation of oil palm has been successfully developed using somatic embryogenesis techniques [16]. However, the physiological characteristics of in vitro seedlings remain poorly understood. Rival et al. [17] conducted one of the first studies on in vitro gas exchange in oil palms, employing an experimental airtight glass chamber to measure physiological variables. According to Sáez et al. [18], the detailed physiological characterization allows a better understanding of the interaction between cultivated species and environmental factors, producing stable plants with minimal genetic variation.

Despite its significance, few studies have documented the effects of in vitro development on oil palm leaf structure and physiology or how these factors affect on-field performance [19,20]. Understanding these physiological responses is essential for refining the acclimatization process and improving plant adaptation and survival.

This study aimed to characterize the physiological changes in in vitro-derived oil palm seedlings from in vitro development through acclimatization and later field establishment. Comparisons were also made between in vitro-derived and seed-derived seedlings. By identifying key physiological and biochemical responses during the acclimatization, we seek to optimize the process, increasing the percentage of successfully acclimated seedlings and ultimately improving the field establishment rates. Additionally, this research will contribute to refining laboratory protocols, improving plant material quality, and expanding the knowledge of adaptation mechanisms in later ex vitro phases, such as nursery and field stages.

## 2. Results

### 2.1. Gas Exchange

**Figure 1:**
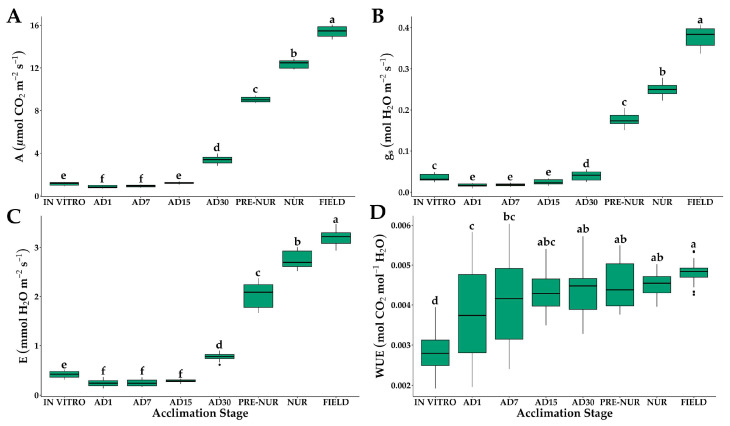
Gas exchange of in vitro-obtained oil palm seedlings at different acclimation stages. (**A**) Net photosynthetic rate (*A*); (**B**) stomatal conductance (*g_s_*); (**C**) transpiration rate (*E*); (**D**) water use efficiency (*WUE*). IN VITRO corresponds to completely developed seedlings before starting the acclimation process. Acclimation stages include days of acclimation 1, 7, 15, and 30 (AD1, AD7, AD15, and AD30), pre-nursery (PRE-NUR), nursery (NUR), and fully acclimated seedlings planted in the field (FIELD). Different letters denote statistically significant differences among stages, as determined by Tukey’s HSD test (*p* < 0.05). n = 30.

Significant differences were observed in gas exchange variables across the different developmental stages of oil palm seedlings (Figure 1). For net photosynthetic rate (*A*), a sharp decline was observed on the first day ex vitro, decreasing from 1.15 μmol m^−2^ s^−1^ (in vitro) to 0.86 μmol m^−2^ s^−1^ at acclimation day 1 (AD1). Recovery began after the first week, with values reaching 3.39 μmol m^−2^ s^−1^ at AD30. Significant increases were observed in the pre-nursery, nursery, and field stages (9.03, 12.39, and 15.4 μmol m^−2^ s^−1^, respectively), indicating improved photosynthetic efficiency as seedlings adapted to ex vitro conditions (Figure 1A). The progressive increment in *A* suggests that leaf development and the activation of the photosynthetic apparatus are key factors in acclimatization.

Stomatal conductance (*g_s_*) exhibited a significant reduction in the first week ex vitro, dropping from 0.034 mol H_2_O m^−2^ s^−1^ (in vitro) to 0.018 mol H_2_O m^−2^ s^−1^ (AD1). Gradual recovery was observed from AD15 onwards, reaching 0.040 mol H_2_O m^−2^ s^−1^ (AD30). The highest values were recorded in the nursery (0.25 mol H_2_O m^−2^ s^−1^) and field stages (0.38 mol H_2_O m^−2^ s^−1^) (Figure 1B). The initial decrease suggests incomplete stomatal regulation during early acclimatization, while the later increase indicates improved stomatal functionality.

Transpiration rates (*E*) followed a similar pattern, with an initial decrease from 0.42 mmol m^−2^ s^−1^ (in vitro) to 0.25 mmol m^−2^ s^−1^ (AD1). A gradual increase was observed from AD7 onward, reaching 3.21 mmol m^−2^ s^−1^ in the field stage (Figure 1C). The lowest values in the early ex vitro stages indicate a protective response to minimize water loss, likely due to incomplete stomatal function. The progressive rise in *E* at later stages reflects the establishment of fully functional stomata, allowing increased gas exchange and evaporative cooling.

Water use efficiency (*WUE*) exhibited an increasing trend as seedlings acclimatized. The ratio of photosynthesis (*A*) to transpiration (*E*) was initially low in in vitro seedlings (0.0028 mol CO_2_ mol^−1^ H_2_O), increasing to 0.0048 mol CO_2_ mol^−1^ H_2_O in the field stage (Figure 1D). This progressive improvement in *WUE* highlights the optimization of gas exchange mechanisms, with seedlings becoming more efficient in carbon assimilation relative to water loss as they transitioned to field conditions.

### 2.2. Light and CO_2_ Response Curve Analyses

**Figure 2:**
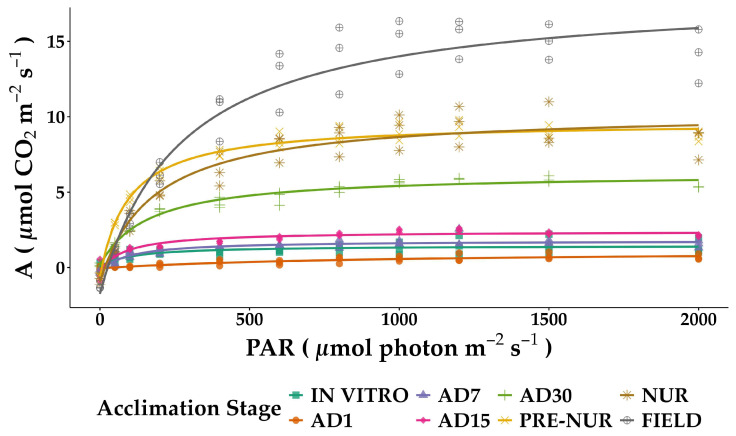
Photosynthetic light response curves of in vitro-derived oil palm seedlings (*Elaeis guineensis*) at various acclimation stages. Net photosynthesis (A) was measured under varying irradiance levels of photosynthetic photon flux density (PAR). IN VITRO corresponds to completely developed seedlings before starting the acclimation process. Acclimation stages include days of acclimation 1, 7, 15, and 30 (AD1, AD7, AD15, and AD30), pre-nursery (PRE-NUR), nursery (NUR), and fully acclimated seedlings planted in the field (FIELD). Curves were fitted using the Michaelis–Menten rectangular hyperbolic function. Each data point represents the individual measurements from multiple replicates at each stage.

Light saturation and CO_2_ response curves demonstrated significant physiological adjustments across analyzed developmental stages. In the case of the light response curves (Figure 2), the maximum photosynthetic rate (*A_max_*) exhibited a clear trend of increasing capacity as plants progressed through the acclimation stages. The lowest *A_max_* values were observed in seedlings obtained in vitro (1.42 ± 0.12 µmol CO_2_ m^−2^ s^−1^), reflecting the limitations of controlled conditions. As plants transitioned to acclimation, *A_max_* showed a moderate increase, reaching 2.64 ± 0.22 µmol CO_2_ m^−2^ s^−1^ in AD15. A more pronounced increase occurred in later stages, with field plants displaying the highest *A_max_* (20.01 ± 0.98 µmol CO_2_ m^−2^ s^−1^), indicating the complete acclimation and optimal carbon assimilation efficiency (Table 1).

The constant of saturation, representing the light intensity required to reach half of *A_max_*, showed substantial variation among acclimation stages. Early-stage plants (in vitro and AD7) exhibited low saturation values (104.18 ± 16.84 and 93.17 ± 10.07 µmol photon m^−2^ s^−1^, respectively), indicating a high photosynthetic response at low irradiance. In contrast, plants in the AD1 stage required much higher light intensities to reach saturation (904.70 ± 148.28 µmol photon m^−2^ s^−1^), suggesting early acclimation constraints. A progressive increase in light saturation was observed in the field stage (269.70 ± 28.84 µmol photon m^−2^ s^−1^), indicating an adaptation to high light availability (Table 1).

The dark respiration rate (*Rd*), which accounts for mitochondrial CO_2_ release in the absence of light, was the lowest in seedlings obtained in vitro (−0.04 ± 0.11 µmol CO_2_ m^−2^ s^−1^). A slight increase occurred in AD7 and AD15 (−0.27 ± 0.11 and −0.23 ± 0.21 µmol CO_2_ m^−2^ s^−1^, respectively), suggesting a progressive adjustment in respiratory activity. The highest *Rd* values were observed in field plants (−1.75 ± 0.79 µmol CO_2_ m^−2^ s^−1^), consistent with an enhanced respiratory demand associated with more significant metabolic activity in fully acclimated plants (Table 1).

The light compensation point (*LCP*), which represents the minimum light required for net CO_2_ assimilation to be positive, followed a decreasing trend during acclimation. In vitro seedlings had an *LCP* of 2.64 µmol photon m^−2^ s^−1^, which remained relatively low in AD30 (3.74 µmol photon m^−2^ s^−1^), suggesting high light-use efficiency under low irradiance. However, an increment was observed in the nursery and field stages (16.09 and 25.86 µmol photon m^−2^ s^−1^, respectively), reflecting a shift towards higher light dependency in fully acclimated plants (Table 1).

The quantum yield of CO_2_ (Φ), estimated as the initial slope of the light response curve, was highest in pre-nursery plants (0.0517 mol CO_2_ mol^−1^ photon), suggesting an optimal capacity to utilize light at low intensities. The lowest Φ values were observed in AD1 plants (0.0009 mol CO_2_ mol^−1^ photon), indicating a temporary limitation in light capture and CO_2_ fixation. A subsequent increase was observed in later stages, with field plants reaching 0.0420 mol CO_2_ mol^−1^ photon, reflecting a well-adjusted light utilization strategy.

**Figure 3:**
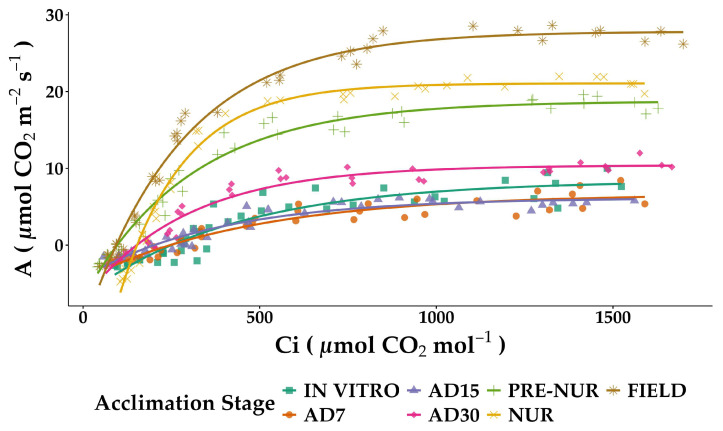
Photosynthetic CO_2_ response curves of in vitro-derived oil palm seedlings (*Elaeis guineensis* Jacq.) at various acclimation stages. Net photosynthesis rate (*A*) was measured under varying external CO_2_ levels. IN VITRO corresponds to completely developed seedlings before starting the acclimation process. Acclimation stages include days of acclimation 1, 7, 15, and 30 (AD1, AD7, AD15, and AD30), pre-nursery (PRE-NUR), nursery (NUR), and fully acclimated seedlings planted in the field (FIELD). The presented curves were fitted using the model described by Tezara et al. (2003) [21]. Each data point represents individual measurements from multiple replicates at each stage and corresponds to specific internal CO_2_ concentrations (C_i_).

The physiological parameters measured from CO_2_ response curves (Figure 3) revealed significant variations during the acclimation process of in vitro oil palm seedlings compared to pre-nursery, nursery, and fully acclimated field-grown plants (Table 2).

The in vitro seedlings exhibited relatively low values for CO_2_-saturated photosynthetic rate (*A_sat_*) at 8.39 ± 1.1 μmol CO_2_ m^−2^ s^−1^ and low carboxylation efficiency (*CE*) at 0.062 ± 0.021 μmol CO_2_ m^−2^ s^−1^. During the initial phase of acclimation (AD7 and AD15), *A_sat_* further decreased slightly to 6.65 ± 1.0 and 6.15 ± 0.3 μmol CO_2_ m^−2^ s^−1^, respectively, with *CE* also decreasing markedly at AD7 (0.026 ± 0.0011) and remaining relatively low at AD15 (0.030 ± 0.0045). However, after 30 days of acclimation (AD30), a notable improvement in physiological performance occurred, with *A_sat_* significantly increasing to 10.40 ± 0.3 μmol CO_2_ m^−2^ s^−1^ and *CE* almost returning to initial values (0.061 ± 0.005).

Progressive acclimation stages from pre-nursery to field conditions showed notable enhancements in all photosynthetic parameters. Pre-nursery plants demonstrated substantial improvements, with *A_sat_* at 18.74 ± 0.1 μmol CO_2_ m^−2^ s^−1^ and *CE* increasing to 0.085 ± 0.0092. Nursery plants further improved, exhibiting an *A_sat_* of 21.07 ± 0.2 μmol CO_2_ m^−2^ s^−1^ and a notable peak in *CE* (0.284 ± 0.007). The fully adapted seedling planted in the field showed the highest physiological performance, with maximum Amax reaching 27.83 ± 0.3 μmol CO_2_ m^−2^ s^−1^ and *CE* stabilizing slightly lower than in nursery plants 0.154 ± 0.021. Similar trends were observed for other critical parameters, including the CO_2_ offset point (*Γ*), which progressively declined from 251.9 ± 28.4 μmol mol^−1^ in vitro to 95.5 ± 8.8 μmol mol^−1^ in field plants. The relative stomatal limitation (*L_s_*) and mesophyll limitation (*L_m_*) also showed progressive decreases through the acclimation stages, with mesophyll limitation reaching negligible values (0.00 ± 1.11) in fully acclimated field plants, indicating the optimized mesophyll functioning.

Key enzymatic activities associated with photosynthesis, including the maximum carboxylation rate of RuBisCO (*V_cmax_*), maximum electron transport rate (*J_max_*), and triose phosphate utilization rate (*TPU*), followed similar patterns, progressively increasing from in vitro through to field plants. Field plants recorded the highest values, *V_cmax_* (91.0 ± 4.7 μmol m^−2^ s^−1^), *J_max_* (125.9 ± 1.8 μmol m^−2^ s^−1^), and *TPU* (9.8 ± 0.2 μmol m^−2^ s^−1^), demonstrating an optimized photosynthetic efficiency at complete acclimation.

### 2.3. Chlorophyll Fluorescence

**Figure 4:**
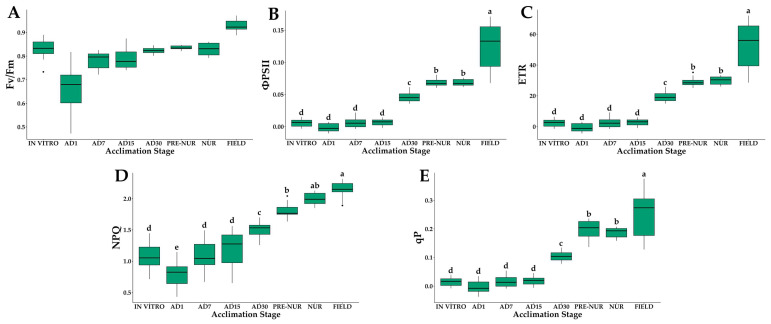
Chlorophyll fluorescence parameters of in vitro-derived oil palm seedlings (*Elaeis guineensis* Jacq.) at various acclimation stages. (**A**) Maximum quantum yield of PSII (*F_v_/F_m_*); (**B**) quantum yield of PSII (*Φ_PSII_*); (**C**) electron transfer rate (*ETR*); (**D**) non-photochemical (*NPQ*); (**E**) photochemical quenching (*q_P_*). IN VITRO corresponds to completely developed seedlings before starting the acclimation process. Acclimation stages include days of acclimation 1, 7, 15, and 30 (AD1, AD7, AD15, and AD30), pre-nursery (PRE-NUR), nursery (NUR), and fully acclimated seedlings planted in the field (FIELD). Different letters denote statistically significant differences among stages, as determined by Tukey’s HSD test (*p* < 0.05). n = 10.

The chlorophyll fluorescence parameters highlighted the physiological stress during early ex vitro stages (Figure 4). The maximum quantum yield of PSII (*F_v_*/*F_m_*) was the lowest in AD1 (0.65), indicating significant photoinhibition. A gradual improvement was observed, with values increasing progressively during acclimatization, reaching stable values in field-planted seedlings (*F_v_*/*F_m_* = 0.83 ± 0.01) (Figure 4A).

The effective quantum yield of photosystem II (*Φ_PSII_*) was lowest at AD1 (0.002 ± 0.007), indicating substantial photochemical limitations at the early stage. *Φ_PSII_* increased progressively through subsequent acclimatization stages, reaching a maximum in the palms planted in the field (0.12 ± 0.04). (Figure 4B).

Electron transport rates (*ETR*) were the highest in field palms (51.8 μmol m^−2^ s^−1^) and the lowest in AD1 (0.71 μmol m^−2^ s^−1^) (Figure 4C).

Non-photochemical quenching (*NPQ*) showed the lowest values in plants in vitro and in AD1 (1.0–0.8, respectively), increasing progressively as the days of acclimatization progressed until it showed the maximum values in the field (Figure 4D). Photochemical quenching (*q_P_*) values progressively increased from 0.016 (in vitro) to 0.25 (field stage) (Figure 4E). These patterns confirm that photoprotective mechanisms were activated during acclimatization to mitigate photoinhibition.

### 2.4. Comparison Between In Vitro- and Seed-Derived Seedlings in the Nursery Stage

A comparison of gas exchange parameters between in vitro-generated seedlings and those derived from seeds in the nursery stage revealed significant differences. Seed-derived palms exhibited slightly higher photosynthesis (11.86 μmol CO_2_ m^−2^ s^−1^) than clonal palms (11.20 μmol CO_2_ m^−2^ s^−1^). Similarly, the transpiration (3.62 vs. 2.78 mmol H_2_O m^−2^ s^−1^) and stomatal conductance (0.31 vs. 0.24 mol H_2_O m^−2^ s^−1^) were higher in seed-derived seedlings. However, in vitro-generated seedlings exhibited greater *WUE* (0.0041 vs. 0.0033 mol CO_2_ mol^−1^ H_2_O) (Table 3). These differences suggest micropropagated seedlings can match seed-derived seedlings in photosynthetic capacity, but their acclimatization and water management strategies differ.

## 3. Discussion

The physiological performance of in vitro-derived oil palm seedlings across different acclimation stages highlights critical adjustments in gas exchange, chlorophyll fluorescence, and photosynthetic capacity. These findings provide insights into the biochemical and physiological constraints limiting the seedling performance during early acclimatization and the subsequent improvements associated with ex vitro adaptation.

Micropropagation presents a viable alternative for the large-scale production of elite oil palm genotypes, but acclimatization remains a major bottleneck affecting plant survival and productivity. Tissue-cultured plants often experience significant physiological stress upon transfer from controlled in vitro environments to external conditions, primarily due to low photosynthetic efficiency, inadequate stomatal function, and altered carbon metabolism [22,23]. The gradual increase in net photosynthetic rate (*A*) across acclimation stages confirms that in vitro-derived oil palm seedlings progressively enhance their photosynthetic capacity as they transition from heterotrophic to autotrophic growth. These observations align with the findings in micropropagated bamboo (*Dendrocalamus hamiltonii*), where gas exchange parameters in seedlings obtained in vitro approached those of seed-derived plants following a successful acclimatization phase [13].

The low *A* observed in vitro and the early acclimation stages are consistent with previous reports describing limited CO_2_ assimilation under in vitro conditions due to high sucrose availability and low internal CO_2_ concentrations [24]. The reliance on externally supplied sugars during in vitro culture can suppress endogenous photosynthesis by downregulating photosynthetic gene expression, reducing rubisco activity, and altering carbon partitioning [25]. While sucrose supplementation is essential for in vitro growth, it can delay the onset of fully functional autotrophy, as observed in *Fragaria x ananassa*, where high sucrose levels reduced photosynthetic enzyme activity and led to a decline in net CO_2_ assimilation [26].

A decrease in *A* at the beginning of the ex vitro phase, followed by recovery, has been reported in multiple species, including *Olea europaea* [9] and *Capsicum annuum*, where photosynthesis values initially declined before increasing past in vitro levels within a month [27]. Similar patterns were observed in *Cocos nucifera* [28] and *Musa AAB* [29], reinforcing that early acclimatization is critical for improving photosynthetic capacity.

The progressive increase in *A* suggests that oil palm seedlings can successfully transition to autotrophic growth, albeit with a prolonged adjustment period. At the nursery stage, photosynthetic capacity had significantly improved, reaching values comparable to those reported for seed-derived palms [20]. These findings reinforce the notion that micropropagated plants, despite initial physiological limitations, can eventually attain photosynthetic rates equivalent to their conventionally propagated counterparts. Similar trends have been reported in clonal oil palms, where photosynthesis values for elite clones remained within 2 µmol CO_2_ m^−2^ s^−1^ of seed-derived plants [20].

In addition to net CO_2_ assimilation, the stomatal function was critical in acclimatization. Stomatal conductance (*g_s_*) remained low in vitro, a common feature of micropropagated plants due to incomplete stomatal development and impaired responsiveness to environmental cues [14]. This limitation can hinder CO_2_ uptake and contribute to the low photosynthetic rates observed in early acclimation. However, as the seedlings progressed through the acclimatization stages, *g_s_* increased significantly, indicating that stomatal function had been restored. A similar recovery was reported in *Asparagus officinalis*, where acclimatization under controlled humidity conditions gradually improved stomatal responsiveness, ultimately enhancing net CO_2_ assimilation [30].

Water-use efficiency (*WUE*) also followed a progressive trend, with significant improvements observed at later acclimation stages. The initially low *WUE* values in in vitro and early ex vitro stages likely reflect excessive water loss due to inefficient stomatal control, commonly reported in tissue-cultured plants [31]. The subsequent increase in *WUE*, particularly in the field-planted palms, suggests that acclimatization resulted in a more efficient balance between carbon gain and water loss. Comparable findings were reported in *Anthurium andraeanum*, where tissue-cultured plants exhibited reduced transpiration rates and improved *WUE* after prolonged acclimatization [32].

The light compensation points observed in this study reflect developmental differences across acclimatization stages. The high light compensation values in early ex vitro stages suggest that seedlings require higher light intensities to achieve net CO_2_ assimilation, as observed in *Nicotiana tabacum* [33]. Similar trends have been noted in multiple species, including *Hevea brasiliensis*, where *F_v_/F_m_* stabilization occurred after 30 days [34]. The progressive reduction in the light compensation point and improvements in photosynthetic efficiency confirm that the acclimatization leads to a functional photosynthetic apparatus.

The CO_2_ response curves highlight the biochemical constraints on photosynthesis during early acclimatization. Carboxylation efficiency (*CE*) and RuBisCO activity improved progressively, with final field-stage values comparable to those reported for seed-grown oil palms [35]. The increasing *CE* values indicate that stomatal and biochemical limitations were gradually overcome, allowing for more efficient CO_2_ fixation. Similar adaptations have been observed in other C_3_ species, where early acclimation stages exhibited limited RuBisCO activity, which improved as plants transitioned to ex vitro conditions [36]. The observed trends suggest that CO_2_ limitations in early stages are gradually alleviated, ensuring efficient carbon assimilation during plant maturation.

Chlorophyll fluorescence parameters provided additional insights into the physiological status of plants during acclimatization. The maximum quantum efficiency of PSII photochemistry (*F_v_/F_m_*) remained lower in the initial acclimatization stages, indicative of photoinhibition and stress during the transition from in vitro to ex vitro conditions. As acclimatization progressed, *F_v_/F_m_* values gradually increased, suggesting the recovery of photochemical efficiency. The highest values were observed in field plants, indicating a fully functional photosynthetic apparatus.

Non-photochemical quenching (*NPQ*) followed the same pattern, with the lowest values detected in the early acclimatization stages. The increase in *NPQ* across later stages suggests that seedlings progressively adjusted their photoprotective mechanisms, increasing excess energy dissipation as their photosynthetic machinery stabilized. The observed trends in photochemical quenching (*q_P_*) and the electron transport rate (*ETR*) further support the conclusion that acclimatization improves photosynthetic performance, enabling plants to fully utilize the available light energy.

The differences in chlorophyll fluorescence parameters between in vitro and seed-derived seedlings in the nursery stage highlight key physiological distinctions. While seed-derived seedlings exhibited slightly higher *F_v_/F_m_* values, indicating better photochemical efficiency, in vitro-derived seedlings displayed comparable *NPQ* values, suggesting effective photoprotective mechanisms. These findings confirm that while in vitro-generated plants may initially experience great stress during the acclimatization process, they can develop functional photochemical and gas exchange properties comparable to their seed-derived counterparts over time, as previously observed in *Gevuina avellana* [37] and *Phalaenopsis* [38].

The direct comparison between in vitro-generated and seed-derived seedlings in the nursery stage provided key insights into potential physiological constraints associated with micropropagation. While the net photosynthetic rate (*A*) was slightly lower in seedlings obtained in vitro than in seed-derived counterparts, significant differences were observed in transpiration and stomatal conductance. Seedlings from seeds exhibited higher *E* and *g*_s_, suggesting a more developed stomatal apparatus and greater water loss regulation capacity. However, in vitro-derived seedlings displayed a higher *WUE*, indicating that these plants could maintain a more favorable carbon gain-to-water loss ratio despite lower *g_s_*.

The patterns observed in gas exchange, chlorophyll fluorescence, and water use dynamics highlight the complex physiological adjustments required to acclimate micropropagated oil palm seedlings successfully. Early-stage limitations in photosynthesis and stomatal conductance underscore the need for controlled environmental transitions to optimize seedling establishment. The progressive enhancement in *A*, *WUE*, and photochemical efficiency suggests that micropropagated seedlings can achieve a photosynthetic performance comparable to seed-derived plants, given adequate acclimatization time and conditions [39].

These findings align with previous studies on other micropropagated species, where acclimatization was crucial for developing fully functional photosynthetic machinery. The balance among CO_2_ uptake, water regulation, and photochemical efficiency during acclimatization plays a vital role in determining the long-term field performance of micropropagated plants. The optimized acclimatization protocols by fine-tuning environmental parameters, such as light intensity and humidity, could further enhance the physiological competence of in vitro-derived oil palm seedlings, improving their field establishment and productivity [40,41].

## 4. Materials and Methods

### 4.1. Location

The study was conducted at the Experimental Field “Palmar de la Vizcaína”, located in Barrancabermeja, Santander, Colombia. The region has an average annual temperature of 32 °C, relative humidity of 70%, an altitude of 125 m above sea level, and an annual rainfall of 2852 mm. The laboratory’s growth room temperatures were 28 °C ± 2 °C, with a relative humidity between 45% and 60% and a 16 h photoperiod. In the greenhouse, temperature conditions were 27 °C ± 2 °C. Seedlings were protected from high temperatures and radiation with two layers of 70% polyshade for the first 15 days, followed by a single layer. Relative humidity ranged from 80% to 40%. The acclimatization phase was conducted in a laboratory and a net house designed to harden micropropagated oil palm seedlings.

### 4.2. Plant Material

The seedlings were obtained from the Plant Tissue Culture Laboratory of Cenipalma (Barrancabermeja, Colombia). Fully expanded leaves were sampled from rooted seedlings at different developmental stages according to their hardening status: in vitro seedlings, corresponding to wholly developed seedlings before starting the acclimation process. Acclimation stages included days of acclimation 1, 7, 15, and 30 (AD1, AD7, AD15, and AD30), pre-nursery (PRE-NUR), nursery (NUR), and fully acclimated seedlings planted in the field (FIELD). Seed-generated palms from commercial nurseries were included for comparison.

### 4.3. Gas Exchange Measurements

Gas exchange measurements were performed on 30 seedlings from each developmental stage: in vitro, AD1, AD7, AD15, AD30, pre-nursery, nursery, and field-grown palms. During the measurement process, in vitro seedlings were temporarily removed from their culture jars, and the most developed leaf was carefully unfolded and placed inside the measurement chamber. A similar procedure was followed for seedlings at different acclimatization stages. For pre-nursery and nursery stages, measurements were taken from the central section of leaf 3, whereas for the in vitro-derived plants already planted in the field, leaflets from 3/5 of leaf 17 were selected.

Gas exchange parameters were recorded using a LI-6800 infrared gas analyzer (IRGA) (LI-COR, Lincoln, NE, USA). The analyzer had removable aperture inserts to adjust the chamber area based on leaf size. Inserts of 3 cm² were used for seedlings in the in vitro and early acclimatization stages, while 6 cm² inserts were used for the pre-nursery, nursery, and field stages.

The measured variables included net photosynthetic rate (*A*), transpiration rate (*E*), and stomatal conductance (*g_s_*). The measurements were conducted between 8:30 and 11:30 AM. Water use efficiency (*WUE*) was calculated as the photosynthesis-to-transpiration ratio. Standard reference conditions were maintained at 400 μmol mol^−1^ of CO_2_ concentration, 1000 μmol m^−2^ s^−1^ of photosynthetic photon flux density (*PPFD*), a water vapor saturation pressure of 2.5 kPa, and a maximum coefficient of variation (CV) of 3%. The average ambient temperature during measurements was 28 °C.

### 4.4. Light and CO_2_ Curves

Light and CO_2_ response curves were generated for each developmental stage. The most developed leaf was selected for in vitro and early acclimatization stages. For pre-nursery and nursery seedlings, the leaf three was sampled, while the leaf 17 was used for field-grown palms.

Light saturation curves were obtained using the “Light_Response” function of the LI-6800. The parameters for these measurements were a temperature of 28 °C, a CO_2_ concentration of 400 μmol mol^−1^, a saturation vapor pressure of 2.5 kPa, and a maximum coefficient of variation of 3%. The program started with a photosynthetic photon flux density (*PPFD)* of 2000 μmol m^−2^ s^−1^ and decreased in increments of 200 μmol m^−2^ s^−1^ until reaching zero to determine the light compensation point.

The photosynthesis response curves to *PPFD* were modeled according to [42], using the rectangular hyperbolic Michaelis–Menten equation:

$$A=\frac{A_{max}*PPFD}{\left(K+PPFD\right)}-R_{d}$$

where

- *A_max_* is the maximum photosynthetic rate;
- *K* is the saturation constant for light (equal to ½ PPFD);
- *R_d_* is the dark respiration rate.

CO_2_ response curves were obtained from three plants per stage using the “CO_2_ Response” function of the LI-6800, starting at a CO_2_ concentration of 400 μmol mol^−1^ and decreasing stepwise by 200 μmol mol^−1^ to zero before progressively increasing to 1800 μmol mol^−1^. The reference conditions were a temperature of 28 °C, a PPFD of 1000 ± 5 μmol m^−2^ s^−1^, a saturation vapor pressure of 2.5 kPa, and a coefficient of variation of 3%. *A/C_i_* curves were fitted using the empirical equation proposed by Tezara et al. [21] for semi-arid ecosystems and later adapted for oil palm [43].

### 4.5. Statistical Analysis

All data were analyzed using a randomized design and processed in R version 4.1.1. For gas exchange measurements, analysis of variance (ANOVA) was conducted, followed by post-hoc comparisons using Tukey’s test.

## 5. Conclusions

This study demonstrates that the successful acclimatization of in vitro-derived oil palm seedlings involves complex and interrelated physiological adjustments. Key physiological processes are progressively optimized throughout the acclimatization phases, including transitions from heterotrophic to autotrophic metabolism, improvements in stomatal functionality, and enhanced water-use efficiency. The recovery of photosynthetic and photochemical parameters during acclimatization ultimately enables seedlings to achieve a performance comparable to that of seed-derived seedlings, confirming the viability of micropropagation as a robust propagation method for elite oil palm genotypes. Furthermore, careful environmental management during acclimatization, particularly concerning irradiance, humidity, and carbon availability, is crucial to minimize the plant stress and to maximize physiological adaptation. Future research should focus on refining specific environmental conditions and management practices during acclimatization to enhance the physiological competence, survival rates, and field performance of micropropagated oil palms, ultimately improving their commercial application and sustainability.

## Figures and Tables

**Table 1:** Photosynthetic parameters derived from light-response curves at various acclimation stages of in vitro-derived oil palm seedlings (*Elaeis guineensis*).

Acclimation Stage	Maximum Photosynthesis (µmol CO_2_ m^−^² s^−^¹)	Saturation Constant (μmol photon m^−2^ s^−1^)	Dark Respiration Rate (μmol CO_2_ m^−2^ s^−1^)	Light Compensation Point (μmol photon m^−2^ s^−1^)	Quantum Yield of CO_2_ (mol CO_2_ mol^−1^ photon)
IN VITRO	1.42 ± 0.12	104.18 ± 16.84	−0.04 ± 0.11	2.64	0.0081
AD1	1.17 ± 0.14	904.70 ± 148.28	−0.05 ± 0.04	39.95	0.0009
AD7	2.05 ± 0.12	93.17 ± 10.07	−0.27 ±0.11	14.01	0.0118
AD15	2.64 ± 0.22	86.93 ± 13.36	−0.23 ± 0.21	8.28	0.0156
AD30	6.11 ± 0.31	160.33 ± 3.24	−0.15 ± 0.28	3.74	0.0229
PRE-NURSERY	10.25 ± 0.27	95.02 ± 4.67	−0.59 ± 0.25	5.80	0.0517
NURSERY	11.30 ± 0.54	177.43 ± 17.19	−0.94 ± 0.49	16.09	0.0399
FIELD	20.01 ± 0.98	269.70 ± 28.84	−1.75 ± 0.79	25.86	0.0420

IN VITRO corresponds to completely developed seedlings before starting the acclimation process. Acclimation stages include days of acclimation 1, 7, 15, and 30 (AD1, AD7, AD15, and AD30), pre-nursery (PRE-NUR), nursery (NUR), and fully acclimated seedlings planted in the field (FIELD). Values correspond to the mean ± SD, n = 3.

**Table 2:** Photosynthetic parameters derived from CO_2_ responsive curves at various acclimation stages of in vitro-derived oil palm seedlings (*Elaeis guineensis* Jacq.).

	A_sat_ (CO_2_-Saturated Photosynthetic Rate) (μmol CO_2_ m^−^² s^−^¹)	CE (Carboxylation Efficiency) (mol CO_2_ m^−^² s^−^¹)	Γ (CO_2_ Compensation Point) (μmol mol^−1^)	L_s_ (Relative Stomatal Limitation) (%)	L_m_ (Mesophyll Limitation) (%)	V_cmax_ (Maximum Carboxylation Rate of RuBisCO) (μmol m^−^² s^−^¹)	J_max_ (Maximum Electron Transport Rate) (μmol m^−^² s^−^¹)	TPU (Triose Phosphate Utilization Rate) (μmol m^−^² s^−^¹)
IN VITRO	8.4 ± 1.1	0.062 ± 0.021	251.9 ± 28.4	66.1 ± 4.7	71.8 ± 4.1	12.0 ± 1.8	35.3 ± 4.8	3.0 ± 0.3
AD7	6.7 ± 1.0	0.026 ± 0.001	246.6 ± 23.5	66.4 ± 3.2	76.6 ± 3. 6	12.6 ± 1.7	29.0 ± 2.5	2.6 ± 0.3
AD15	6.2 ± 0.3	0.030 ± 0.004	202.9 ± 16.6	55.4 ± 5.3	77.8 ± 1.0	12.8 ± 1.3	34.3 ± 1.5	2.5 ± 0.1
AD30	10.4 ± 0.3	0.061 ± 0.005	152.6 ± 9.1	38.2 ± 2.8	62.4 ± 1.2	26.2 ± 2.1	53.7 ± 2.5	4.0 ± 0.1
PRE-NURSERY	18.7 ± 0.1	0.085 ± 0.009	97.7 ± 7.7	28.5 ± 2.1	32.1 ± 0.5	53.3± 4.9	87.5 ± 3.3	6.7 ± 0.1
NURSERY	21.1 ± 0.2	0.284 ± 0.007	153.2 ± 7.2	32.0 ± 1.9	24.1 ± 0.6	53.1 ± 4.5	105.8 ± 0.6	7.6± 0.1
FIELD	27.8 ± 0.3	0.154 ± 0.021	95.5 ± 8.8	26.6 ± 0.8	0.00 ± 1.1	91.0 ± 4.7	125.9 ± 1.8	9.8± 0.2

IN VITRO corresponds to completely developed seedlings before starting the acclimation process. Acclimation stages include days of acclimation 1, 7, 15, and 30 (AD1, AD7, AD15, and AD30), pre-nursery (PRE-NUR), nursery (NUR), and fully acclimated seedlings planted in the field (FIELD). Values correspond to the mean ± SD, n = 3.

**Table 3:** Photosynthetic characteristics of in vitro and seed-generated oil palm nursery seedlings.

Parameter	In Vitro Generated Seedlings	Seed Generated Seedlings
Photosynthesis (µmol CO_2_ m^−2^ s^−^¹)	11.20 ± 0.61 b	11.86 ± 0.72 a
Transpiration (mmol H_2_O m^−2^ s^−^¹)	2.78 ± 0.39 b	3.62 ± 0.50 a
Stomatal conductance (mol H_2_O m^−2^ s^−^¹)	0.24 ± 0.02 b	0.31 ± 0.04 a
Water Use Efficiency (mole CO_2_ mol^−1^ H_2_O)	0.0041 ± 0.0006 a	0.0033 ± 0.0005 b

Values correspond to the mean ± SD. Different letters denote statistically significant differences among stages, as determined by Tukey’s HSD test (*p* < 0.05), n = 30.

## Data Availability

Data are available on request from the authors.
